# Essential genes identification model based on sequence feature map and graph convolutional neural network

**DOI:** 10.1186/s12864-024-09958-w

**Published:** 2024-01-10

**Authors:** Wenxing Hu, Mengshan Li, Haiyang Xiao, Lixin Guan

**Affiliations:** College of Physics and Electronic Information, Gannan Normal University, Ganzhou, Jiangxi 341000 China

**Keywords:** Essential genes, Graphical convolutional neural networks, Machine learning, Gene sequences, Bioinformatics

## Abstract

**Background:**

Essential genes encode functions that play a vital role in the life activities of organisms, encompassing growth, development, immune system functioning, and cell structure maintenance. Conventional experimental techniques for identifying essential genes are resource-intensive and time-consuming, and the accuracy of current machine learning models needs further enhancement. Therefore, it is crucial to develop a robust computational model to accurately predict essential genes.

**Results:**

In this study, we introduce GCNN-SFM, a computational model for identifying essential genes in organisms, based on graph convolutional neural networks (GCNN). GCNN-SFM integrates a graph convolutional layer, a convolutional layer, and a fully connected layer to model and extract features from gene sequences of essential genes. Initially, the gene sequence is transformed into a feature map using coding techniques. Subsequently, a multi-layer GCN is employed to perform graph convolution operations, effectively capturing both local and global features of the gene sequence. Further feature extraction is performed, followed by integrating convolution and fully-connected layers to generate prediction results for essential genes. The gradient descent algorithm is utilized to iteratively update the cross-entropy loss function, thereby enhancing the accuracy of the prediction results. Meanwhile, model parameters are tuned to determine the optimal parameter combination that yields the best prediction performance during training.

**Conclusions:**

Experimental evaluation demonstrates that GCNN-SFM surpasses various advanced essential gene prediction models and achieves an average accuracy of 94.53%. This study presents a novel and effective approach for identifying essential genes, which has significant implications for biology and genomics research.

## Introduction

Essential genes, which are currently a hot topic in genomics and bioinformatics research, are indispensable for supporting cellular life [1]. Their coding functions are crucial for the survival of organisms. These genes constitute a set that must be present in an organism and are vital for maintaining its life activities under specific environmental conditions. They encode key proteins or RNA molecules that are essential for life, and their functions are considered fundamental for the organism's survival [2]. In humans and other organisms, the functions of essential genes are often associated with basic cellular metabolism, growth, development, the immune system, and the maintenance of cellular structure. Therefore, the study of essential genes is of great importance for our understanding of the fundamental physiological functions of organisms and the mechanisms of disease occurrence [3, 4].

With the completion of whole-genome sequencing and the development of genome-scale gene inactivation techniques, it has become possible to identify essential genes within the genome. Traditional experimental techniques used to identify essential genes [5] in organisms include gene knockout [6, 7] and gene silencing [8]. Gene knockout is the process of inactivating a specific gene in an organism to observe its effects on the organism's survival and function. This can be accomplished through various techniques, including CRISPR-Cas9 gene editing [9]. The aim of knockout is to determine whether a gene is an essential gene, that is, whether the absence of the gene would make the organism non-viable. On the other hand, gene silencing is used to study the function of a gene by interfering with or suppressing its expression, often accomplished through methods such as RNA interference [10] and antibodies [11]. However, these traditional experimental methods still have several potential drawbacks: they are expensive, time-consuming, and do not offer comprehensive genome coverage. In modern biological research, machine learning models have been developed to computationally identify essential genes [12]. These methods have been extensively employed to study essential genes and contribute to advancing our understanding of gene function and organismal complexity.

In machine learning methods for predicting essential genes, feature extraction is a key step that involves extracting useful feature information from genomic data for model learning. This feature information is combined with machine learning classification algorithms (SVM [13], NB [5, 13–15], RF [13, 16], etc.) to build models for essential gene prediction. High-throughput genome sequencing and homology localization [17] provide a variety of biological features for predicting essential genes, including network topology information [18, 19], homology information [20, 21], gene expression information [22, 23], and functional domains [23]. For instance, Deng et al. developed an integrated classifier for essential genes by integrating information from diverse features extracted from different aspects of the essential genome sequence [24]. Chen and Xu also successfully combined high throughput data with machine learning methods to determine protein deficiencies in Saccharomyces cerevisiae [25]. Seringhaus et al. used various intrinsic genomic features to train machine learning models to predict essential genes in brewer's yeast [26], and Yuan et al. developed three machine learning methods to predict lethality in mouse knockouts based on informative genomic features, among others [27]. However, these data are often not available [28, 29], and some data features do not have high predictive power or even add biological redundancy. Consequently, there are also models currently being constructed based on DNA sequence features of essential genes [30]. For instance, Ning et al. employed single nucleotide frequencies, dinucleotide frequencies, and amino acid frequencies of gene sequences to predict essential genes in bacteria [31]. Guo et al. emphasized the significance of local nucleotide composition and internal nucleotide association, proposing an approach known as λ-interval Z-curve to integrate both types of information [32]. Chen et al. combined Z-curve pseudo-k-tuple nucleotide composition with an SVM classifier to construct a model aimed at capturing DNA sequence patterns associated with essential genes [33]. In addition to these methods, Le et al. utilized natural language processing methods to comprehend DNA sequence features associated with gene essentiality and integrated deep neural networks to predict these essential genes [34], Rout et al. conducted feature counting, including parameters such as energy, entropy, uniformity, and contrast within nucleotides [35], while simultaneously employing supervised machine learning methods for identification, among other techniques.Overall, there is a growing body of research utilizing machine learning methods for essential gene prediction [36, 37], which has led to significant improvements in prediction performance. However, Most machine learning methods for predicting essential genes rely on their protein sequence data. The fundamental principle is that the importance of such genes is determined by the absence of functional roles played by their protein products. Considering that nucleotide-based features have not been thoroughly explored, our work aims to utilize the inherent information within nucleotides. We seek to explore new research methodologies and unearth the significant impact of gene sequences in predicting essential genes, thereby enhancing the recognition performance of the model.

While machine learning-based approaches successfully predict essential genes, they exhibit significant variations in terms of the methods used for sequence feature extraction and the employed model structures. The predictive performance of a method relies on its ability to explore gene feature information and integrate it into the model structure effectively. Thus, enhancing model performance is critical in investigating novel methods. In this context, the primary contribution of this study lies in proposing and applying an innovative sequence feature graph encoding method that effectively translates genetic sequence information into the graph structure representation required by deep learning models. Initially, gene sequences are transformed into a set of subsequences containing k nucleotides each. Through the statistical analysis of these subsequence frequency data and the relationships between adjacent subsequences, a graph structure representing the features of gene sequences is constructed. This encoding method not only overcomes the complexity of the original sequences but also offers an effective means to capture essential genetic sequence information, thereby laying the foundation for subsequent applications of deep learning models. Furthermore, this study introduces an innovative model framework based on Graph Convolutional Neural Networks (GCNN), namely GCNN-SFM. This model combines graph convolutional layers, convolutional layers, and fully connected layers to effectively learn and utilize both local and global information within the sequence feature graph. GCNN-SFM not only captures the intricate features of gene sequences but also enhances the accuracy and robustness of gene prediction tasks. Through the design of this model structure, we successfully applied Graph Convolutional Neural Networks to the essential gene prediction task in the field of bioinformatics, offering new insights and methods for research in this domain. Beyond the innovative application of the model framework, this study fine-tuned model parameters and utilized gradient descent algorithms to optimize the model's loss function, significantly contributing to enhancing the model's performance and predictive accuracy. Overall, this research presents a novel and effective deep learning method for essential gene analysis and prediction tasks, offering critical insights for related studies in the field of bioinformatics.

## Theory and computational section

### Datasets

In bioinformatics research, generalized benchmark datasets are crucial for constructing high-performance predictive models. In this study, we utilized datasets from four species: *Drosophila melanogaster* (D.melanogaster), *Methanococcus maripaludis* (M.maripaludis), *Caenorhabditis elegans*(C.elegans [38]), and *Homo sapiens* (H.sapiens). These datasets represent highly comprehensive resources in this specific field. Campos et al. curated comprehensive genomic data and associated annotations for D.melanogaster from sources such as FlyBase (http://ftp.flybase.net/genomes/Drosophila_melanogaster/) [39], Ensembl databases (https://ftp.ensembl.org/pub/current_fasta/drosophila_melanogaster/) [40], and peer-reviewed journal articles [41]. Similarly, data for C.elegans were collected from WormBase (https://wormbase.org/species/c_elegans#1402--10) [42], Ensembl databases (https://ftp.ensembl.org/pub/current_fasta/caenorhabditis_elegans/), and peer-reviewed journal articles [43]. Chen et al. [33] obtained the complete genome of M.maripaludis from the DEG (Database of Essential Genes: https://tubic.org/deg/public/index.php) [44], a comprehensive repository encompassing all available essential gene information. To reduce data redundancy and mitigate homology bias, sequences exhibiting over 80% structural similarity were excluded from the DEG. Furthermore, gene data for H. sapiens were extracted from the DEG database by Guo et al. [32]. Therefore, this paper selected the datasets defined by the aforementioned individuals, which encompass both positive and negative datasets of essential genes. The benchmark dataset can be represented as:

1 $${\mathbb{S}}= {\mathbb{S}}^{+} \cup {\mathbb{S}}^{-}$$

Where ${\mathbb{S}}$ represents the entire dataset for a particular species, ${\mathbb{S}}^{+}$ denotes the positive subset of essential genes, and ${\mathbb{S}}^{-}$ denotes the negative subset of essential genes. The union of these two subsets is defined as $\cup$.The provided dataset was divided into three sets: a training set, a validation set, and a test set, with a ratio of 8:1:1. The validation set assesses the model's generalization ability and detects overfitting during training, while the test set evaluates the model's performance after the completion of training. The details of the datasets are presented in Table 1.

**Table 1:** Number of gene sequences in the datasets

Dataset	Train set		Verification set		Test set		Reference
	Positive	Negative	Positive	Negative	Positive	Negative	
D. melanogaster	1628	3797	249	580	313	731	[41]
H.sapiens	2873	6702	319	745	401	935	[32]
M. maripaludis	414	857	46	95	58	120	[33]
C.elegans	3688	8604	743	1733	1108	2586	[43]

### *Gapped k-mer* encoding feature extraction

The model predicts essential genes by encoding the gene sequence into the matrix format required for deep learning. Features are extracted from the gene sequence of an essential gene using *Gapped k-mers *[45, 46] encoding, resulting in a graph structure. In the field of bioinformatics, *k-mers* refer to subsequences of length k that are found within gene sequences. To transform a sequence into numerical representations, it is necessary to generate k-mers by sliding a fixed-size window of length k. During this process, the DNA sequence is fragmented into subsequences, referred to as k-mers, each representing a set of nucleotides. The size of a k-mer or subsequence depends on the window size used to generate them. For example, in Table 2, a sequence with a length of L can be divided into *L-k* + *1 k-mers*, depending on the value of *k*.

**Table 2:** *k-mer* for gene sequence

Gene sequence: GTACTA	
*k*	*k-mer*
1	G,T,A,C,T,A
2	GT,TA,AC,CT,TA
3	GTA,TAC,ACT,CTA
4	CTAC,TACT,ACTA
5	GTACT,TACTA
6	GATCTA

To address the genetic variation that often occurs in biological sequences, in this study, we specifically examine bases that are separated by a distance of d unrelated positions within the sequences. Referring to Table 2, the subsequence GTA can be represented as GT $$**$$ A when *k* = *3* and *d* = *3*, with $$*$$ representing the allowable distance within the gene sequence. After segmenting the sequence into multiple k-mers, we compute the occurrence frequency of each nucleotide group within these k-mers. These frequencies are then extracted to construct a graphical vector, which serves as the input. Specifically, the gene sequence is initially partitioned into various nucleotide groups based on k-mers. Frequencies are computed for each k-mer group and the occurrence of adjacent k-mer groups. Subsequently, these k-mer groups, based on the sequence of bases in the gene, are connected to form a graph structure. Equation (1) is employed to represent the structure of the graph.

1 $$G=\left(n, e\right)$$

**Fig. 1:**
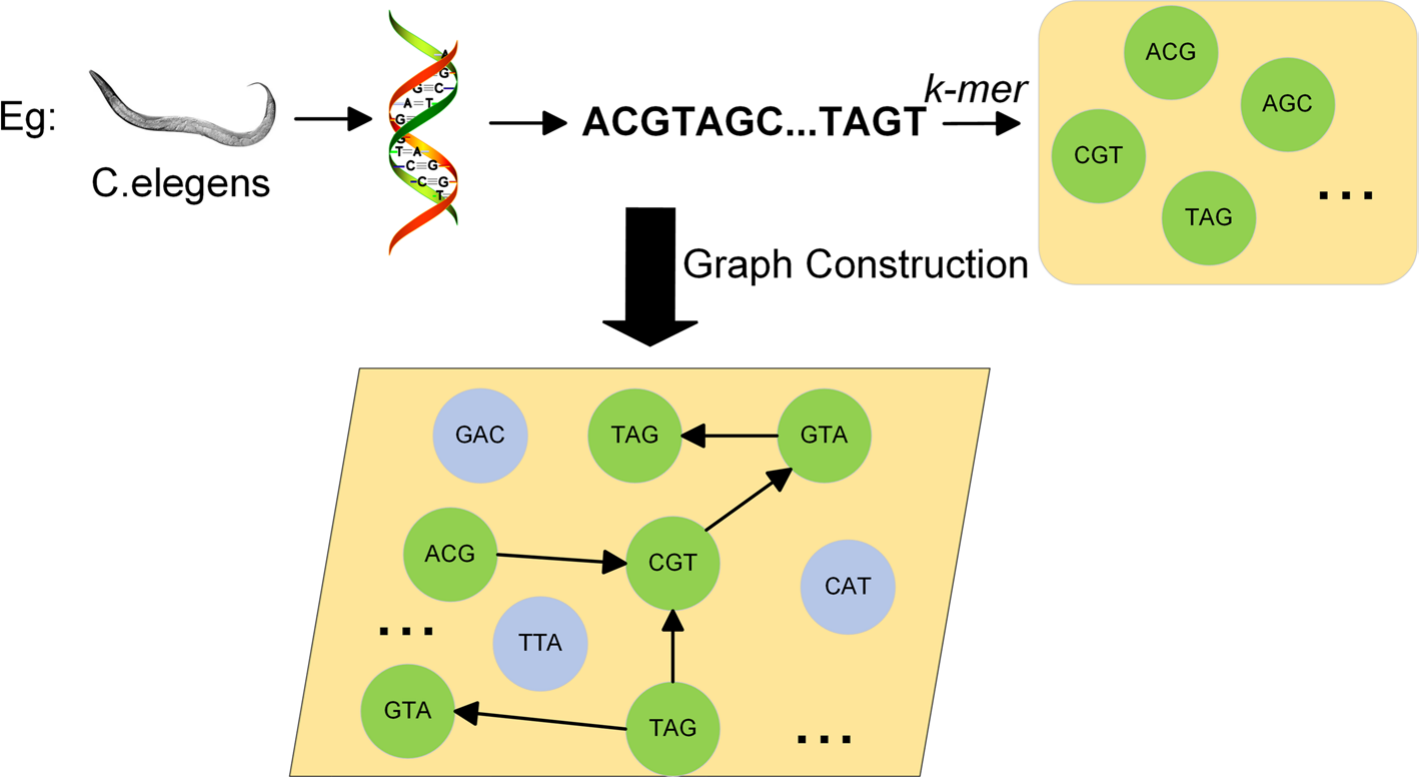
Graph structure of gene sequences

Where $n$ represents the set of nodes, while $e$ represents the set of connected edges. The characteristic information of each node is determined by the frequency of occurrence of its respective *k-mer*, whereas the characteristic information of a connected edge is determined by the frequency of occurrence of two *k-mers* together. When *k* = *3*, as illustrated in Fig. 1, the gene sequence of the essential gene can be transformed into a graph structure.The generated sequence feature graph represents the occurrence frequency of k-mers within the gene sequence, as well as the connectivity between these k-mers. This representation serves as input for subsequent deep learning models.

### Models based on sequence feature maps and graph convolutional neural networks

**Fig. 2:**
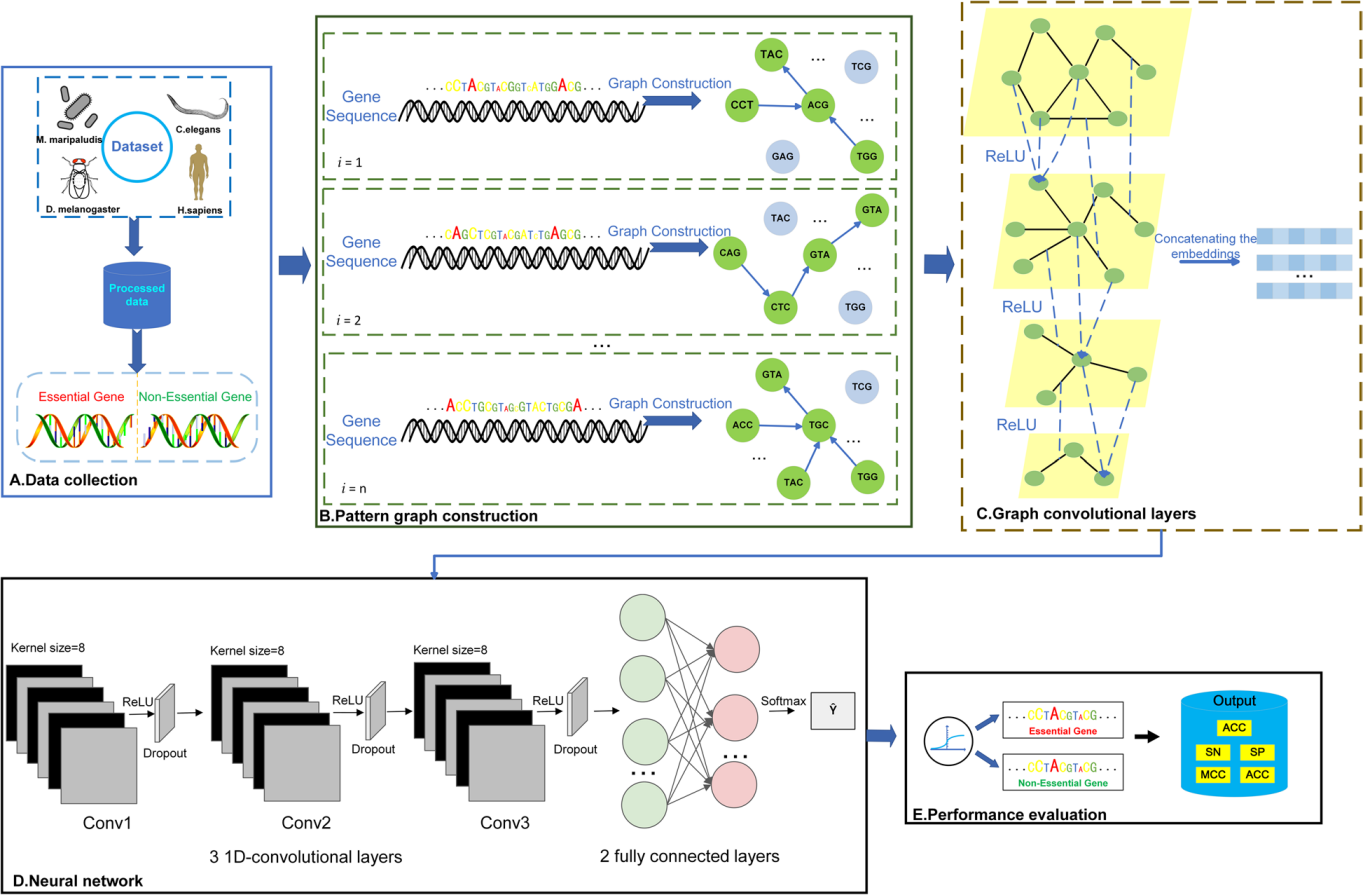
Model GCNN-SFM predicts the structural flow of essential genes

In this study, we adopt a multi-layered Graph Convolutional Neural Network (GCNN) structure, abbreviated as GCNN-SFM, aiming to conduct feature learning and prediction on the sequence feature graph. The primary objective is to address feature learning and prediction tasks using this model structure. After applying the above encoding scheme, the gene sequences are transformed into graph structures. Graph Convolutional Neural Network is a deep learning model capable of processing graph data to perform feature learning and prediction tasks. Unlike traditional convolutional neural networks (CNNs), graph convolutional neural networks can handle irregular graph data with arbitrary connectivity relationships. The core components of the GCNN-SFM model are as follows: Firstly, the Graph Convolutional Layers serve as the primary foundation of GCNN-SFM. Consisting of four graph convolutional layers, this segment aims to update and aggregate node feature information. Each graph convolutional layer comprises two critical steps: neighbor node feature aggregation and feature transformation. During the neighbor node feature aggregation phase, the model aggregates features of the nodes within each graph convolutional layer, considering the connections between nodes and their feature similarities, to compute weights for updating node representations. Subsequently, in the feature transformation step, the model conducts linear transformations and non-linear activation operations on the features post neighbor node feature aggregation, aiming to acquire higher-order and more expressive node representations. Lastly, the GCNN-SFM model employs three one-dimensional convolutional layers to further extract features and maps node representations to the label space of the prediction task using fully connected layers. This process aims to accomplish the prediction task on gene graph data, facilitating effective identification and prediction of essential genes. The design of the GCNN-SFM model structure aims to fully leverage the advantages of Graph Convolutional Neural Networks in handling graph-structured data. Through successive processing, aggregation, and transformation, it achieves deeper feature learning from sequence feature graphs and accurate execution of prediction tasks.The structure of GCNN-SFM is depicted in Fig. 2.

In the graph convolutional layers of the GCNN-SFM model, the aggregation of neighboring node features stands as a crucial and pivotal step. This step aims to aggregate information from the neighbors of node ${v}_{i}$ by considering the connections between nodes and the similarity of their features, weighted by specific weights. This process computes a completely new representation for each node. The formulation for the feature aggregation process is represented as Eq. (2):

2 $${Z}_{i}^{(k)}={\sum }_{j\in N(i)}\frac{1}{\sqrt{{d}_{i}{d}_{j}}}\cdot {h}_{j}^{\left(k-1\right)}\cdot {W}^{\left(k-1\right)}$$

Where ${Z}_{i}^{(k)}$ represents the aggregated features of node ${v}_{i}$ at the k-th layer, $N\left(i\right)$ is the set of neighboring nodes of node ${v}_{i}$, ${d}_{i}$ and ${d}_{j}$ are the degrees of nodes ${v}_{i}$ and ${v}_{j}$ respectively, ${h}_{j}^{\left(k-1\right)}$ stands for the features of node ${v}_{j}$ at the (k-1)-th layer, and ${W}^{\left(k-1\right)}$ is the weight matrix utilized for conducting linear transformations on features.

In each graph convolutional layer, the features of neighboring nodes are aggregated based on the connectivity and feature similarities between nodes. This process aims to effectively leverage the connection structure and feature information among nodes, integrating and fusing the features of neighboring nodes via weighted aggregation. Such an approach aims to update and enhance the representation of each node comprehensively. This updating process provides the GCNN-SFM model with richer and more effective node representations, forming the foundation for feature learning and prediction tasks.

The feature transformation step is one of the crucial elements within the graph convolutional layers. Following the aggregation of neighboring node features, node representations are updated through a sequence involving linear transformations and nonlinear activation functions. This process aims to enhance node representations by subjecting the aggregated features to linear transformations and subsequent nonlinear activation, thereby achieving higher-dimensional and more expressive node representations. Specifically, the feature transformation process can be described by Eq. (3):

3 $$\left\{\begin{array}{c}{H}_{i}^{\left(k\right)}=\sigma \left({Z}_{i}^{\left(k\right)}\right)\\ ReLU=max(0,x)\end{array}\right.$$

Where ${H}_{i}^{(k)}$ represents the representation matrix of node ${v}_{i}$ at layer k, and $\sigma$ denotes the nonlinear activation function, specifically referring to ReLU in this context.By applying weighted aggregation and nonlinear transformation to the neighboring nodes, the new feature representation ${H}_{i}^{(k)}$ of the current layer's node can be obtained. The GCNN-SFM employs multi-layer graph convolution operations to progressively aggregate and propagate information from the node's neighbors, enriching its feature representation. Subsequently, the node representations are passed into the convolutional layers for additional extraction and processing, reshaping them into a tensor 'x' that aligns with the input shape. Finally, it is fed into a fully connected layer to be mapped to the label space of the prediction task, as demonstrated in Eq. (4).

4 $$\widehat{y}=softmax\left(ReLU\left({W}_{1}\cdot x+{b}_{1}\right)\cdot {W}_{2}+{b}_{2}\right)$$

Where $\widehat{y}$ represents the predicted gene label by the model,  ${W}_{1}$ and ${b}_{1}$ refer to the weight matrix and bias vector of the first fully connected layer. Similarly, ${W}_{2}$ and ${b}_{2}$ represent the weight matrix and bias vector of the second fully connected layer, respectively.

To establish this mapping, it is necessary to define a loss function that measures the discrepancy between the predicted labels and the true labels. This loss function is iteratively updated using gradient descent to minimize the loss and enhance the accuracy of the predictions made by the GCNN-SFM. In this study, the selected loss function is the widely employed cross-entropy loss, commonly used in multi-classification problems.

5 $$\mathcal{L}==-\frac{1}{N}{\sum }_{n=1}^{N}\left({y}^{(n)}\mathit{log}{p}^{(n)}+(1-{y}^{\left(n\right)}) \mathit{log}(1-{p}^{(n)})\right)$$

Where $N$ is the sample size, ${y}^{(n)}$ is the binary variable, and ${p}^{(n)}$ is the probability that the neural network predicts the nth sample as an essential gene.

## Model performance evaluation

To evaluate the classification performance of the model, we employ several commonly used metrics, consistent with the approach taken by Le et al. [34]. These metrics encompass sensitivity (SN), specificity (SP), accuracy (ACC), Matthew correlation coefficient (MCC), and area under the receiver operating characteristic curve (AUC).For ease of comparison, the F1-Score is also introduced here.The specific calculation procedures for each metric are outlined below.

6 $$\left\{\begin{array}{c}SN=\frac{TP}{TP+FN} \times 100\%\\ SP=\frac{TN}{TN+FP} \times 100\%\\ ACC=\frac{TP+TN}{TP+FN+TN+FP} \times 100\%\\ MCC=\frac{TP\times TN-FP\times FN}{\sqrt{\left(TP+FP\right)\times \left(TP+FN\right)\times \left(TN+FP\right)\times (TN+FN)}} \\ AUC=\frac{\sum_{i\in pos}{rank}_{i}-\frac{{num}_{pos}\left({num}_{pos}+1\right)}{2}}{{num}_{pos}{num}_{neg}}\\ F1-Score=\frac{2\times PRE\times SN}{PRE+SN}, PRE=\frac{TP}{TP+FP}\end{array}\right.$$

Among them, TP, TN, FP and FN represent the number of samples whose prediction results are true positive, true negative, false positive and false negative, respectively. The AUC (Area Under Curve) is defined as the area under the ROC curve, enclosed by the coordinate axes. The closer the AUC value is to 1.0, the better the model's performance.

## Results and discussion

### Experimental results for different parameters of sequence coding

**Fig. 3:**
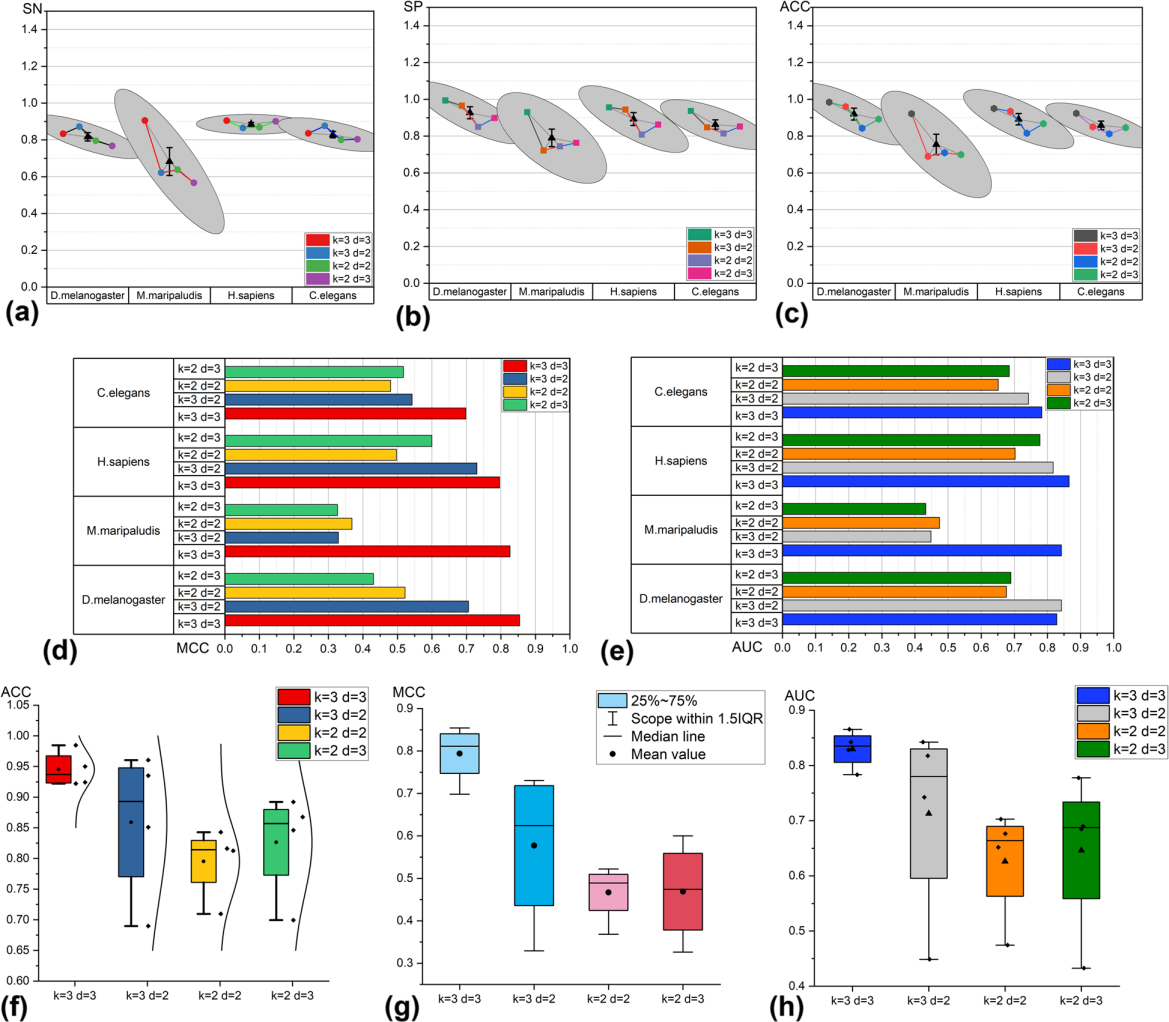
Comparison of performance results of independent datasets testing graph coding methods with different parameters

In most machine learning and deep learning tasks, the encoding method plays a crucial role in obtaining high-quality models. The parameters *k* and *d* in the sequence coding method determine the quality of the sequence feature map. To identify the optimal parameter combination, we conducted preliminary experiments on the data. We combined various values of *k* and *d*, and for each parameter combination, we applied tenfold cross-validation on training and validation sets of four species to determine the best-performing model on the validation set. Subsequently, the model identified as the best performer in the cross-validation task (the model corresponding to a specific parameter combination) was evaluated on the test set. This approach allows validation of the model's generalizability to unseen data and confirms the superiority of the selected parameter. The relevant information of the used dataset is shown in Table 1 and the results obtained are presented in Fig. 3.

Firstly, to determine the optimal parameter settings for the graph coding method and achieve accurate prediction of essential genes, we defined various parameter combinations (*k* = *2, d* = *2; k* = *2, d* = *3; k* = *3, d* = *2; k* = *3, d* = *3*) that were likely to yield optimal performance. Setting the parameters *k* and *d* too high can result in overfitting of the trained model. Figure 3(b) and (c) demonstrate that when both *k* and *d* are set to 3, the model predicts higher values of specificity (SP) and accuracy (ACC) compared to other parameter combinations for essential genes across the four species. The sensitivity (SN) value for M. maripaludis species in Fig. 3(a) is significantly higher, reaching 90%, compared to the other three parameter combinations. These findings suggest that the graph coding method with parameters set to (*k* = *3, d* = *3*) enables more efficient learning of DNA sequence features for essential genes by the model. From Fig. 3(g) and (h), it is evident that the model integrated with the graph coding method using parameter (*k* = *3, d* = *3*) outperformed other parameter combinations, achieving the highest performance across all datasets, with an average accuracy (ACC) of 94.53% and an area under the curve (AUC) of 82.99%. These findings indicate that utilizing the graph coding method with parameters set to (*k* = *3, d* = *3*) enables a more accurate representation of gene sequence characteristics, resulting in superior predictive performance of the model.

### Ablation experiments

**Fig. 4:**
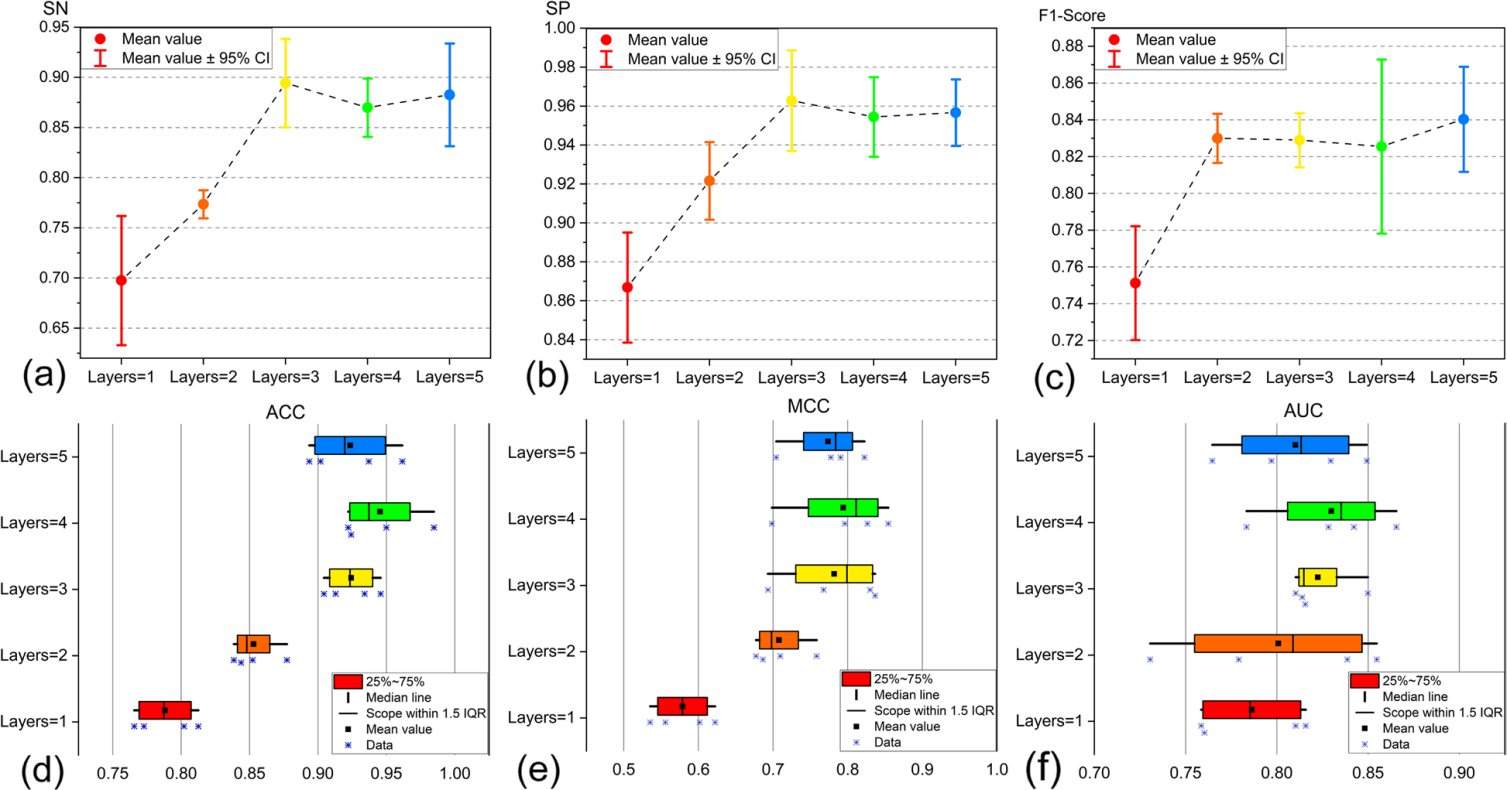
The impact of graph convolutional layer depth on model performance metrics

To explore the influence of the depth of graph convolutional layers on the overall performance of the model, we conducted ablation experiments. Initially, we gradually increased the number of graph convolutional layers from 1 to 5, aiming to elucidate the specific impact of varying graph convolutional layer depths on the performance of the GCNN-SFM model. This was done to determine the most suitable model structure for essential gene identification. The experiments were conducted using datasets from four species, and the obtained evaluation results are illustrated in Fig. 4.

Through ablation experiments, a better understanding of the role of graph convolutional layers in the model and the impact of each layer depth on information extraction and feature learning can be achieved. As depicted in Fig. 4(d), with the increase in the depth of graph convolutional layers, the evaluation metric, ACC, gradually increases. This indicates an improved accuracy of essential gene identification with an increase in the depth of graph convolutional layers. The ACC value peaks at 4 layers, reaching an average value of over 94%. Similarly, MCC and AUC values demonstrate analogous trends. This upward trend reflects the enhancement in the model's classification performance and its improved ability to distinguish samples more accurately. Figure 4(c) illustrates the F1-Score of the model in identifying essential genes. The F1-Score, a harmonic mean of PRE and SN, comprehensively considers both SN and PRE, making it suitable for evaluating scenarios with significant differences in the quantity of samples between different classes. It is evident that the F1-Score reaches over 85% at the 4-layer depth of graph convolutional layers. The fluctuation in model performance might be attributed to overfitting issues in deep graph convolutional networks. An excessive increase in the depth of graph convolutional layers could overly complicate the model, leading to poorer performance.The aforementioned experiments indicate that the model's robustness is highest when employing four layers of graph convolutional layers, providing a reliable basis for further optimizing the model structure.

### Experimental results for different datasets

**Fig. 5:**
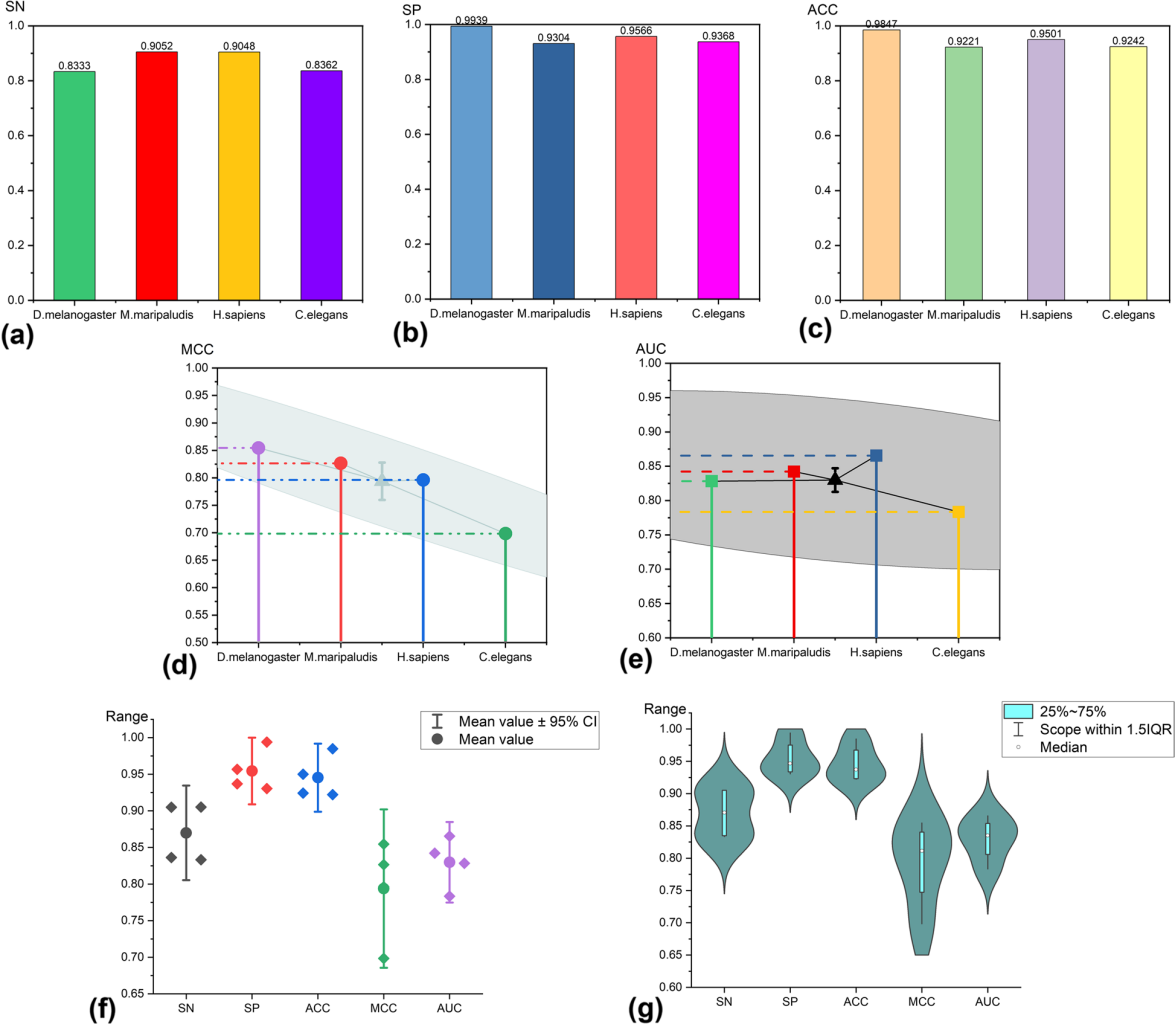
Performance results of different independent datasets testing the essential gene prediction model

To assess the performance of our proposed model GCNN-SFM, we conducted experiments using independent datasets from four species (D.melanogaster, M.maripaludis, H.sapiens, C.elegans) to assess its stability. Based on the results of previous experiments, the model outperformed other parameter combinations when the graph coding method was set to (*k* = *3, d* = *3*). Hence, we selected (*k* = *3, d* = *3*) as the optimal parameter configuration for subsequent experiments. The models underwent training and validation through a tenfold cross-validation process using the training dataset. Prior to this, the DNA gene sequences were transformed into feature matrices using coding methods to facilitate the training and validation of the deep learning models. The trained models were then tested and evaluated on independent test sets, and the predictive performance of each independent dataset is illustrated in Fig. 5.

The GCNN-SFM model exhibited excellent performance for various species, as shown in the experimental results depicted in Fig. 5. Notably, Fig. 5(c) illustrates that the ACC values for predicting essential genes using the model surpassed 90% for all four species, with the D.melanogaster species achieving an exceptionally high ACC value of 98.47%. This finding affirms the validity of the essential gene prediction model. Conversely, in the case of the C. elegans species, as observed in Fig. 5(d) and (e), lower MCC and AUC values were noted compared to those of other species, yet a maintained ACC value of 92.42% was observed. Upon analyzing the SN values, it is hypothesized that the marginally lower MCC and AUC values observed for the C.elegans species result from the limited availability of essential gene data specific to C.elegans. Overall, the model demonstrated remarkable performance across the four species, as illustrated in Fig. 5(f) and Table 3, attaining an average ACC value of 94.53%. These results underscore the stability and reliability of our method, validating its effectiveness as a powerful tool for essential gene prediction.

**Table 3:** Prediction of experimental results for different species and mean values

Dataset	SN	SP	ACC	MCC	AUC
D.melanogaster	0.8333	0.9939	0.9847	0.8545	0.8283
M.maripaludis 4mC_F.vesca	0.9052	0.9304	0.9221	0.8265	0.8422
H.sapiens	0.9048	0.9566	0.9501	0.7961	0.8655
C.elegans	0.8362	0.9368	0.9242	0.6983	0.7834
Average	0.8699	0.9544	0.9453	0.7939	0.8299

### Results of cross-species validation experiments

**Fig. 6:**
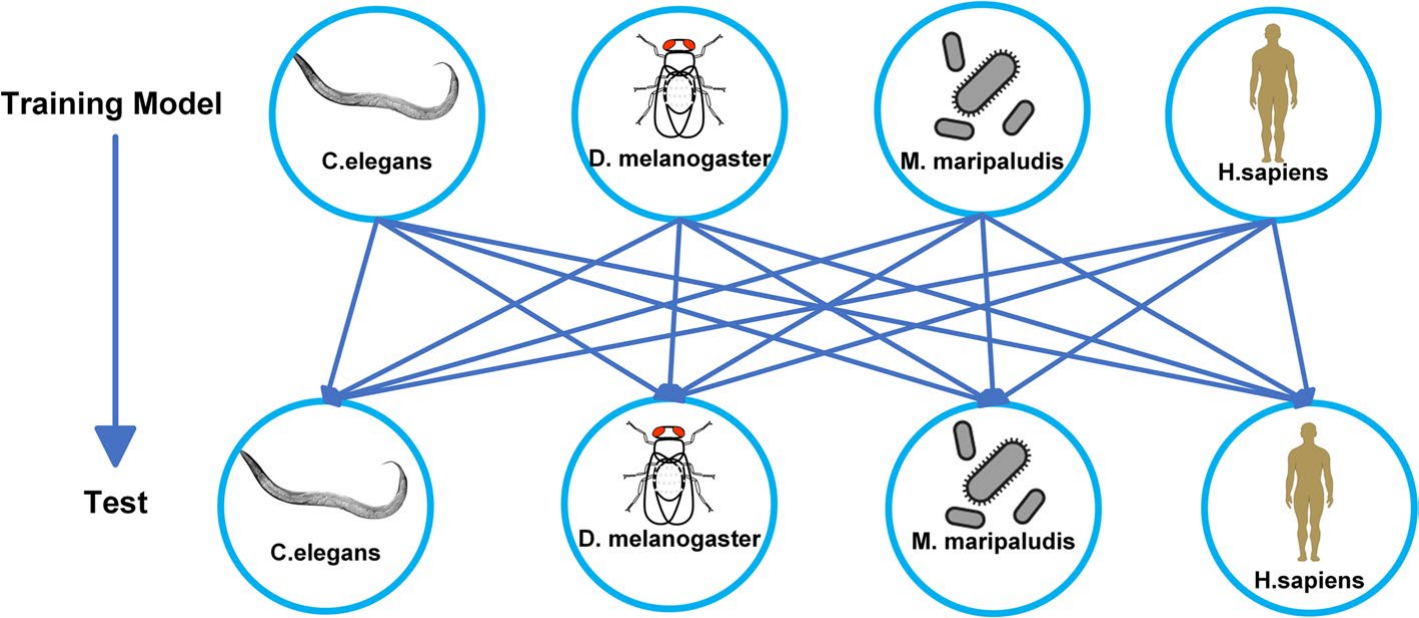
Cross-training of datasets from different species

To investigate whether the DNA sequences of essential genes exhibit specific characteristics or sequence similarities across species, we conducted cross-species validation experiments. This is shown in Fig. 6.

**Fig. 7:**
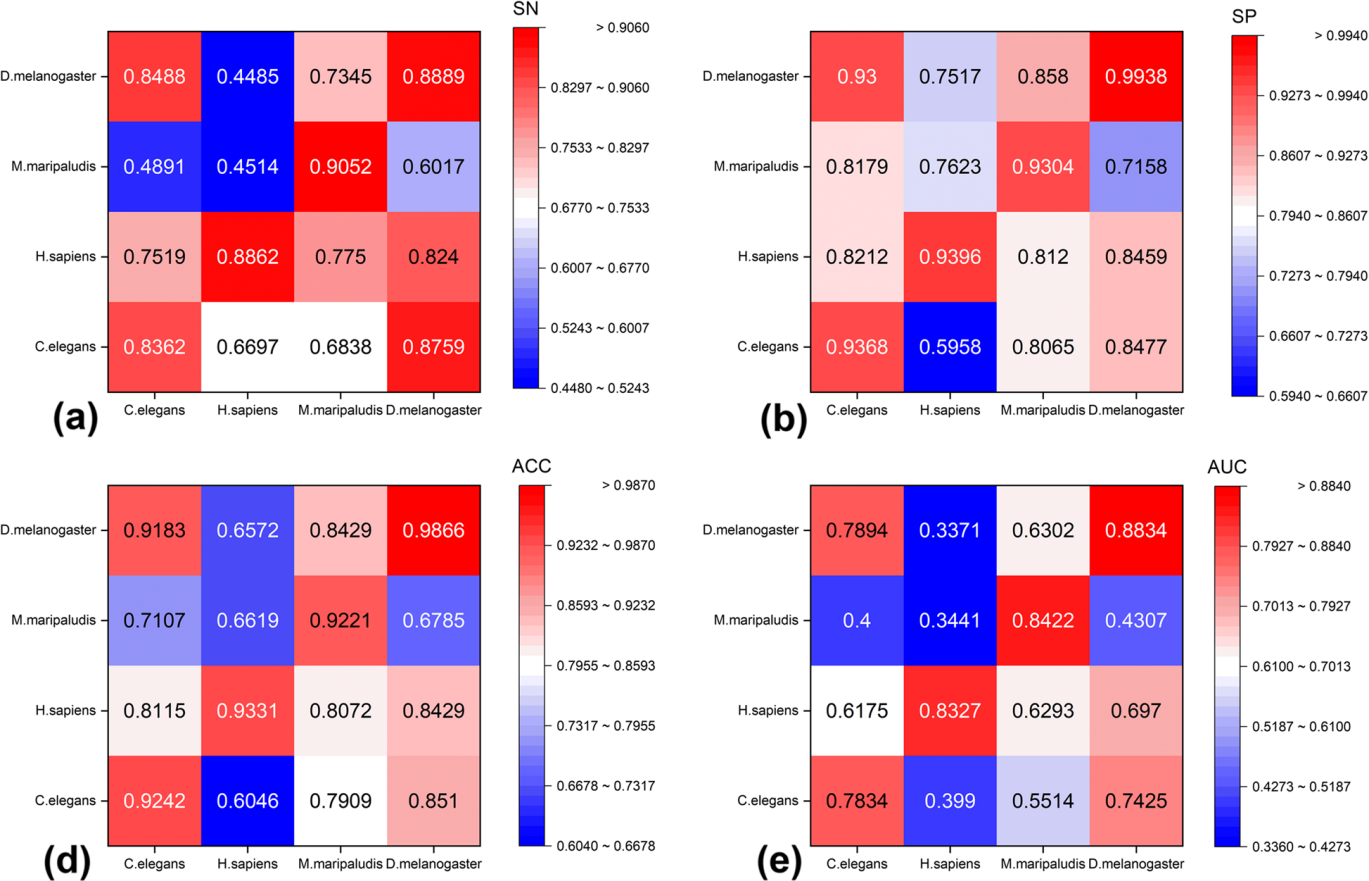
Performance comparison of model validation across species

Using independent datasets from four species (D.melanogaster, M.maripaludis, H.sapiens, and C.elegans), we trained the DNA gene sequences of one species and evaluated the DNA gene sequences of another species to predict whether they were essential genes. The obtained results are depicted in Fig. 7, where the horizontal axis represents the training set, and the vertical axis represents the test set.

Figure 7(d) demonstrates the high accuracy (ACC) observed in two species: D.melanogaster and C.elegans. Training the model with a dataset from the species C.elegans and testing it with D.melanogaster resulted in a model prediction accuracy of 91.83% (ACC). Similarly, training the model with a dataset from D.melanogaster and testing it with C.elegans yielded predictions with an ACC value of 85.1%, suggesting a comparable pattern of nucleotide distribution between the two species. D.melanogaster, C.elegans, M.maripaludis, and H.sapiens exhibited low values for SN, ACC, and AUC, signifying substantial differences in nucleotide distribution among these species. These findings align with the genetic similarity results reported by Campos et al. [47], indicating striking similarities in nucleotide patterns among essential genes in certain species.

### Experimental results comparing performance with other existing methods

To evaluate the effectiveness of our proposed model GCNN-SFM in identifying essential genes, we conducted a comparison with published models that address the same problem. Table 4 displays the pertinent information for each of the compared models.

**Table 4:** Information on each comparison model

Model	Description	Dataset	Reference
Pheg	Combining Z-curve and nucleotide composition learning features for k-intervals using SVM as a classifier	M.maripaludis	[32]
iEsGene-ZCPseKNC	Combining Z-curve and pseudo-k-tuple nucleotide composition learning features using SVM as a classifier	M.maripaludis	[33]
eDNN-EG	Natural language processing model learning features, integrating supervised learning models	M.maripaludis	[34]
IDF-EG	Compute features like energy, entropy, uniformity, contrast, etc., from nucleotides using supervised machine learning	D.melanogaster	[35]
PEG-ML	combines flux balance analysis (FBA) with machine learning	D.melanogaster	[48]
PEGI	Using machine learning methods based on intrinsic gene sequence properties (statistical and physicochemical data)	D.melanogaster	[49]
GCNN-SFM	Gapped k-mer encodes sequences into graph features, combined with graph convolutional neural networks	-	-

**Fig. 8:**
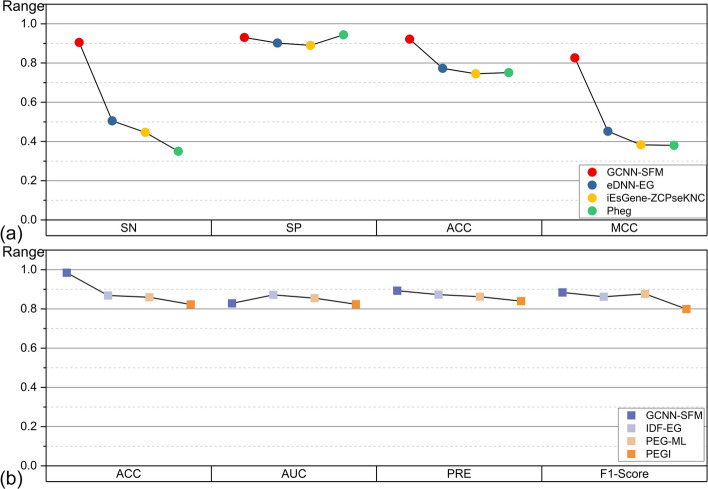
Performance comparison of GCNN-SFM with other existing models

We conducted experiments separately on datasets from the same species used in each model. Due to variations in evaluation metrics among different models, the models using the same standard will be compared separately. The predictive evaluation results of all comparisons are illustrated in Fig. 8.

As shown in Fig. 8(a), GCNN-SFM outperforms eDNN-EG, iEsGene-ZCPseKNC, and Pheg models. Compared to these models, GCNN-SFM exhibits increased ACC values of 14.89%, 17.67%, and 17.09%, respectively. While the SN values of eDNN-EG, iEsGene-ZCPseKNC, and Pheg are significantly lower than their corresponding SP values, GCNN-SFM achieves a higher SN value of 90.52%. The SP value of GCNN-SFM does not differ significantly from that of the other models. The lower SN values of eDNN-EG, iEsGene-ZCPseKNC, and Pheg can be attributed to the considerable imbalance between the numbers of essential gene samples and non-essential gene samples in each training cycle. To address this imbalance, our GCNN-SFM model exclusively employs a sample class weighting strategy during the cross-validation process, preventing overfitting. Consequently, our model achieves an SN value that closely approximates the SP value during prediction.In the comparison depicted in Fig. 8(b), GCNN-SFM exhibited the highest ACC value, reaching 96.45%, surpassing the other three models. Additionally, it demonstrated a higher PRE value. Regarding the evaluation of F1-Score, GCNN-SFM achieved 88.42%. These results demonstrate that the GCNN-SFM model enhances the accuracy of predicting essential genes and outperforms other existing prediction methods.

## Conclusions

This study proposes a graph convolutional neural network (GCNN)-based approach for essential gene prediction. The model GCNN-SFM effectively captures and learns local and global features in gene sequences through graph modeling and feature extraction, enabling the accurate identification of essential genes. The experimental results demonstrate significant performance advantages of our approach in tasks related to essential gene prediction. Our approach excels at extracting more discriminative feature representations in genes compared to traditional methods that rely on sequence feature engineering. Furthermore, this study unveils the potential of GCNN in predicting essential genes, thereby offering a new pathway for comprehending gene function and disease pathogenesis at a deeper level. There are some important considerations to address in future research. Firstly, the model may encounter computational challenges when dealing with large-scale genomic datasets, requiring further optimization and acceleration for practical applications. Secondly, the accuracy of the gene annotation information of the GCNN-SFM model is crucial and has a significant impact on the prediction performance. Numerous studies have employed machine learning methods for protein structure prediction or modeling [50–52]. Future research could further advance and broaden this field, such as integrating multimodal data sources, combining nucleotide data from essential genes with protein data, such as gene expression data and protein interaction networks [15, 53, 54], to enhance the prediction accuracy and robustness. In summary, this study offers robust support for further exploring gene regulatory networks and mechanisms of related diseases by enhancing our understanding of gene function and the prediction of essential genes.

## Data Availability

A static version of the package D.melanogaster and C.elegans datasets containing data linked to this publication is available at: (https://doi.org/10.6084/m9.figshare.12061815) and (https://doi.org/10.6084/m9.figshare.11533101). Thecodes, dataset, architecture, parameters, functions, usage and output of the proposed model are available free of charge at GitHub. (https://github.com/xing1999/GCNN-SFM).
